# Think before prescribing oral contraceptive pills – pulmonary embolism in an obese hypertensive woman: a case report

**DOI:** 10.1097/MS9.0000000000004878

**Published:** 2026-04-06

**Authors:** Syeda Rafiza Sultana, Muhammad Shoaib Momen Majumder, Tarikul Hamid, Mohammad Ali, Anindita Das Barshan, Mohammad Jahid Hasan

**Affiliations:** a Department of Obstetrics and Gynecology, Shaheed Suhrawardy Medical College Hospital, Dhaka, Bangladesh; b Department of Rheumatology, Bangladesh Medical University (Ringgold ID 74464), Dhaka, Bangladesh; c Department of Critical Care Medicine, Ibn Sina Medical College Hospital, Dhaka, Bangladesh; d Pi Research & Development Center, Dhaka, Bangladesh; e Tropical Disease and Health Research Center, Dhaka, Bangladesh

**Keywords:** alteplase, deep vein thrombosis, hypertension, low-dose oral contraceptive pills, obesity, pulmonary embolism

## Abstract

**Introduction and importance:**

This case highlights the importance of suspicion of pulmonary embolism (PE) even after low-dose combined oral contraceptive pills (LD-OCPs) and early diagnosis and prompt thrombolytic therapy administration, especially in resource-constrained settings such as Bangladesh.

**Case presentation:**

A 40-year-old female of Asian origin, Class I obese (BMI 32 kg/m^2^), presented to the emergency department with acute severe dyspnea, pleuritic chest pain, and palpitations. Her medical history was notable for obesity and hypertension, which had been managed with antihypertensive medication for the past 3 years. Additionally, she had been taking a LD-OCP for 4 months for menstrual irregularity. Despite initial management with low-molecular-weight heparin and supportive measures, the patient’s condition rapidly deteriorated to severe cardiogenic shock, necessitating urgent intervention. Thrombolytic therapy with alteplase was administered, which significantly improved the patient’s hemodynamic status. Follow-up imaging revealed a reduction in the thrombus burden and resolution of deep vein thrombosis.

**Clinical discussion:**

This case highlights the critical role of early diagnosis and prompt thrombolytic therapy, such as alteplase, in managing acute PE. Rapid intervention can prevent severe complications. Clinicians must maintain high suspicion in symptomatic patients, especially women on hormonal therapy, and use timely diagnostics like D-dimer and CT angiography.

**Conclusion:**

In resource-limited settings, the timely administration of thrombolytic agents such as alteplase can significantly improve patient outcomes, as demonstrated in this case.

## Background

Pulmonary embolism (PE) is a leading cause of cardiovascular morbidity and mortality worldwide. Globally, acute massive PE affects an estimated 1–2 per 1000 individuals annually, with a mortality rate of approximately 8% even after treatment^[^1^]^. In contrast, untreated patients can have mortality rates as high as 30%, underscoring the critical need for timely diagnosis and intervention^[^2^]^. The burden of PE is particularly pronounced in resource-limited settings, such as Bangladesh, where access to advanced diagnostic tools and treatment options such as thrombolytic therapy may be restricted. Although low-dose contraceptives are widely available as over-the-counter medications in Bangladesh, awareness of the associated thromboembolic risks remains limited. Recent meta-analyses indicate that 33.21% of Bangladeshi women use oral contraceptive pills (OCPs), with many opting for combination estrogen-progestin formulations^[^3^]^. In addition, the increase in sedentary lifestyles and urbanization has contributed to increasing obesity rates in Bangladesh, further increasing the risk of thromboembolic events in this population. This case report discusses the successful management of a 40-year-old woman who developed severe cardiogenic shock due to massive PE while on low-dose oral contraceptive pills (LD-OCPs). This case underscores the importance of careful risk stratification when selecting contraceptive methods and the need for early recognition of PE symptoms to improve patient outcomes. Additionally, this study highlights the life-saving potential of administering tissue plasminogen activator (alteplase) in resource-constrained settings, demonstrating the critical role of timely thrombolytic therapy in managing acute massive PE.

HIGHLIGHTS

- Low-dose oral contraceptive pills (LD-OCPs), even in short-term use, can precipitate life-threatening pulmonary embolism (PE) in women with existing risk factors like obesity and hypertension.
- Early and timely administration of thrombolytic therapy such as alteplase should be considered for survival in massive PE, especially in resource-limited settings
- This case underscores the need for family planning counselling and individualized risk stratification before prescribing OCPs.

## Case presentation and diagnosis

A 40-year-old female of Asian origin presented to the emergency department with acute severe dyspnea, pleuritic chest pain, and palpitations. Notably, she had experienced exertional palpitations for 2 days prior to the onset of these acute symptoms. She denied any recent lower limb discomfort, swelling, fever, rash, joint pain, surgery, or immobilization history. Her medical history included obesity (body mass index 32 kg/m^2^) and hypertension, which were managed with amlodipine 10 mg and losartan 50 mg for 3 years. Additionally, she had been taking a LD-OCP (combination of drospirenone 3 mg and ethinylestradiol 0.030 mg) for intermenstrual bleeding for the past 4 months, as prescribed by her gynecologist. Upon initial examination, the patient appeared to be in significant respiratory distress. Her vital signs were as follows: respiratory rate of 34 b/min, heart rate of 130/min, SpO_2_ of 86%, and hypotension with a blood pressure of 84/50 mm Hg. Her Glasgow Coma Scale score was 12, indicating a reduction in consciousness. A general physical examination revealed no signs of visible venous engorgement, edema, or cyanosis. Chest auscultation was unremarkable, and systemic examinations were normal.

A bedside electrocardiogram revealed T wave inversions in leads V1–V4. Serial measurements of troponin-I indicated myocardial injury, with an initial value of 0.215 ng/mL (reference ≤0.04 ng/mL), peaking at 0.714 ng/mL within 6 hours. With a provisional diagnosis of acute myocardial infarction, the patient was admitted to the coronary care unit and treated with supplemental oxygen (5 L/min), a loading dose of 300 mg of aspirin and clopidogrel each, and subcutaneous enoxaparin 60 mg. Heart rate control was achieved with diltiazem and ivabradine, which later led to bisoprolol substitution.

**Figure 1.**
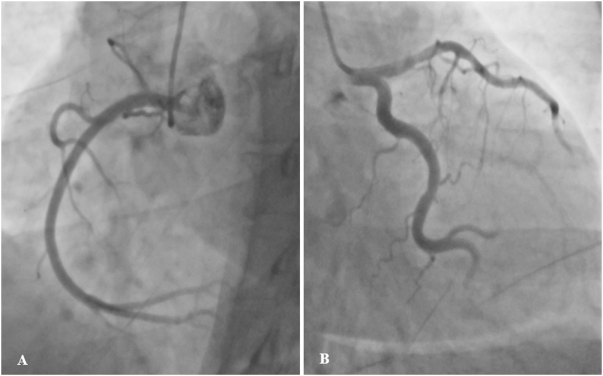
(a) Coronary angiogram (CAG) showing normal right coronary arteries (RCAs), and (b) normal left main, anterior descending, and circumflex arteries.

The following day, a coronary angiogram revealed almost normal coronary arteries, ruling out significant coronary artery disease (Fig. 1a and b). Routine laboratory tests, including complete blood count, liver function tests (ALT), renal function tests (creatinine), and electrolytes, were within normal limits, except for an elevated C-reactive protein level of 31.5 mg/L (reference range <9.1 mg/L for females). The patient was newly diagnosed with diabetes mellitus on the basis of a random blood sugar of 13.8 mmol/L and an HbA1c of 6.6%.

Initially, the patient’s condition stabilized, and she was discharged on subcutaneous enoxaparin (60 mg), aspirin, bisoprolol, telmisartan, ivabradine, and insulin. However, on the sixth day postdischarge, she developed significant exertional dyspnea and palpitations, which led to her readmission on the eighth day.

At the time of readmission, she presented with severe dyspnea and a bedside SpO_2_ of 84%. An echocardiogram revealed right atrial and ventricular dilation, moderate tricuspid regurgitation, and a pulmonary artery systolic pressure (PASP) of 85 mm Hg, indicating severe pulmonary hypertension. Additionally, the right pulmonary artery appeared hazy at its origin, raising suspicion of PE.

Laboratory investigations revealed an elevated D-dimer level of 1450 ng/mL, indicative of significant fibrin degradation, along with a troponin-I level of 0.018 ng/mL (reference <0.034 ng/mL) and an NT-proBNP level of 9940 pg/mL (reference <125 pg/mL), which is consistent with right ventricular strain. Arterial blood gas analysis revealed type I respiratory failure, with a PaO_2_ of 10.47 kPa (reference range 10.5–13.5 kPa), a PaCO_2_ of 3.47 kPa (reference range 5.1–5.6 kPa), and a pH of 7.46 (reference range 7.38–7.42).

**Figure 2.**
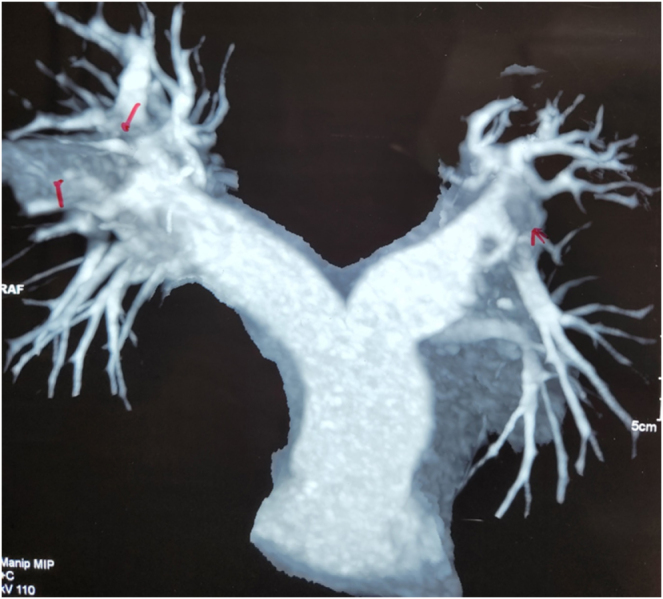
CTPA showing multifocal hypodense structures in the distal right pulmonary and left pulmonary arteries with osteo-proximal obliteration of the left lobar branch, indicating multifocal PE in both pulmonary arteries.

A CT pulmonary angiogram (CTPA) confirmed the diagnosis of multifocal PE, revealing filling defects in both the right and left main pulmonary arteries extending into the left middle lobar branch (Fig. 2). A lower limb duplex ultrasound was performed to investigate the source of the emboli, confirming the presence of acute deep vein thrombosis (DVT) in the right popliteal vein with minimal signs of recanalization. A chest X-ray revealed a small pleural effusion on the right side without any signs of consolidation. Additional laboratory tests, including thyroid function tests (TSH, FT4), electrolytes, calcium, magnesium, and urinalysis, were normal. Thrombophilia screening, including fibrinogen, antithrombin-III, protein C and S activity, and tests for antiphospholipid syndrome (lupus anticoagulant, anticardiolipin, and beta-2 glycoprotein antibodies), yielded no abnormalities.

This case illustrates the critical importance of recognizing the clinical features of PE, especially in the context of risk factors, even the use of LD-OCP, obesity, and hypertension, and the urgent need for timely intervention to prevent fatal outcomes.

## Treatment and outcomes

Despite supplemental oxygen and pharmacological interventions, the patient’s tachycardia, tachypnea, and hypotension persisted, suggesting the onset of severe cardiorespiratory failure. The clinical team recognized the immediate threat of mortality associated with acute massive PE, particularly in light of these worsening signs. Standard anticoagulation therapy, such as subcutaneous enoxaparin or heparin infusion, is insufficient for this critical presentation. At this point, we thoroughly screened the patient for any contraindication to thrombolysis, specifically active internal bleeding, history of hemorrhagic stroke, recent ischemic stroke, central nervous system neoplasm, and recent major trauma or surgery. In addition, advanced interventions such as surgical embolectomy or percutaneous catheter-directed treatments were also unavailable in our resource-limited setting. Given the absence of these contraindications and the unavailability of advanced procedure, the decision was made to administer thrombolytic therapy with alteplase, a potent fibrinolytic agent, to rapidly dissolve the pulmonary arterial clot burden.

The patient was closely monitored in the intensive care unit with continuous assessment of vital signs, arterial blood gases, coagulation parameters, and the potential occurrence of any side effects. Shortly after the initiation of alteplase, the patient exhibited significant improvements in respiratory distress, oxygenation, and blood pressure. Once her hemodynamic status stabilized, follow-up echocardiography revealed almost normal right ventricular function and a normal PASP (22 mm Hg). Upon completion of alteplase infusion, the patient was transitioned to anticoagulation therapy with enoxaparin. A follow-up duplex ultrasound of the lower limbs demonstrated the resolution of DVT in the popliteal artery, confirming successful thrombolysis. Her blood pressure was subsequently managed according to standard hypertension guidelines, maintaining stable readings. Further laboratory investigations, including extensive thrombophilia screening, revealed no significant abnormalities.

## Discussion

This case report describes a 40-year-old female patient who developed massive PE with associated DVT, culminating in cardiogenic shock. This case highlights the critical importance of maintaining a high degree of suspicion regarding possible risk factors, such as the use of LD-OCPs, especially in patients with preexisting conditions such as obesity and hypertension.

While she had been on antihypertensive medication and classified as obese for several years, the recent initiation of LD-OCP raised a strong suspicion of a possible link to the PE event. These factors significantly increased her risk of thromboembolism, which could have been mitigated with a thorough risk assessment prior to prescribing the OCP. Estrogen in OCPs is known to increase the risk of venous thromboembolism (VTE) by creating a hypercoagulable state. It does so by increasing the production of coagulation factors (II, VII, IX, and X) while reducing the levels of natural anticoagulants and inhibiting fibrinolysis through the upregulation of plasminogen activator inhibitor-1^[^4^]^. Compared with nonusers, combined OCPs are associated with a 3.5-fold increased risk of venous thrombosis. The risk is particularly high within the first 4 months of OCP use, as observed in our patient^[^5^]^. Understanding the temporal correlation between the duration of OCP usage and the development of thrombosis is crucial, as it guides the need for vigilant monitoring and risk assessment. Additionally, there is a notable distinction in the risk of thromboembolism between high-dose and low-dose estrogen OCPs. Studies have repeatedly demonstrated that, compared with patients receiving higher-dose formulations (e.g., 50 µg), those receiving a dose equivalent to 30 µg of ethinyl estradiol have a reduced risk of VTE^[^6^]^. A previous study reported an adjusted hazard ratio of 1.23 (95% CI, 0.88–1.73) for VTE with 30 µg of ethinyl estradiol, with the risk varying by age group and being highest in those aged 35–50 years.

Considering other risk factors, obesity and hypertension are two well-established contributors that significantly increase the risk of VTE, encompassing both DVT and PE, through multiple mechanisms^[^7^]^. Obesity, in particular, has been shown to increase the risk of VTE up to six-fold compared with healthy individuals. The highest incidence of VTE has been observed in patients aged 50 years or older, particularly in those with Class II and III obesity^[^8^]^. Although hypertension is not a direct cause of PE, it contributes to endothelial dysfunction and venous stasis, both of which can exacerbate thrombus formation. Given these risks, healthcare providers must recognize the heightened potential for PE when prescribing OCPs, especially in individuals with preexisting conditions such as obesity and hypertension. Non-hormonal contraceptive methods, such as barrier methods or intrauterine devices, may be preferable in these patients as they do not exacerbate the prothrombotic condition. Risk stratification and careful consideration of contraceptive options are essential for preventing life-threatening complications.

Our case report also underscores the importance of early diagnosis and timely initiation of anticoagulation or thrombolytic therapy, such as alteplase, which can significantly reduce the likelihood of severe complications, including right heart failure, stroke, and mortality. Alteplase, a tissue plasminogen activator, binds to fibrin and converts plasminogen to plasmin, leading to the degradation of thrombi. The rapid improvement in the patient’s respiratory and hemodynamic status following thrombolysis highlights the effectiveness of this intervention. A previous meta-analysis of eleven trials demonstrated that thrombolytic therapy is associated with a marked reduction in recurrent PE or death in hemodynamically unstable patients^[^9^]^. A high index of suspicion is essential when managing patients who present with acute symptoms such as exertional palpitations, breathlessness, and chest pain, particularly those with underlying risk factors. The role of low or high doses of OCP or hormonal drugs must be considered in the case of women of reproductive age. Timely diagnostic measures, especially D-dimer testing and CTPA, are crucial in establishing a prompt diagnosis and guiding management.

In terms of prevention, our case report points out an urgent need for low-cost, accessible biomarkers for the prediction of VTE in OCP users, especially in resource-constrained environments where conventional hereditary thrombophilia screening is frequently unaffordable. The normalized Activated Protein C sensitivity ratio (nAPCsr) assay presents a promising possible solution. According to recent economic models, nAPCsr-based screening could prevent up to 13 500 VTE cases a year, thereby saving 1.5 billion euros in medical expenses^[^10^]^.

Previous case reports have also documented the successful treatment of obstructive shock due to acute massive PE with alteplase infusion. Fortunately, our patient did not experience any bleeding or other adverse effects associated with alteplase infusion.

## Conclusion

The successful treatment of this 40-year-old woman highlights the critical importance of timely thrombolytic therapy with alteplase, even in resource-limited settings such as Bangladesh. Moreover, the consideration of LD-OCPs as a potential risk factor, either independently or compounded with other conditions such as obesity and hypertension, should be integral during diagnosis and management. This case emphasizes the need for heightened awareness and vigilant risk assessment when prescribing OCPs (either low-dose or high-dose), as well as a multidisciplinary approach for managing high-risk patients.

## Data Availability

All data are available to the lead author and can be found upon reasonable request from the corresponding author.
